# Sodium selenite penta­hydrate, Na_2_SeO_3_·5H_2_O

**DOI:** 10.1107/S1600536813028602

**Published:** 2013-10-23

**Authors:** Kurt Mereiter

**Affiliations:** a Institute of Chemical Technology and Analytics, Vienna University of Technology, Getreidemarkt 9/164SC, A-1060 Vienna, Austria

## Abstract

In the crystal structure of Na_2_SeO_3_·5H_2_O [disodium selen­ate(IV) penta­hydrate], two Se, two selenite O atoms and one water O atom are located on a mirror plane, and one water O atom is located on a twofold rotation axis. The coordination of one Na^+^ cation is distorted trigonal bipyramidal, formed by three equatorial H_2_O ligands and two axial selenite O atoms. The other Na^+^ cation has an octa­hedral coordination by six water mol­ecules. The two independent SeO_3_ groups form almost undistorted trigonal pyramids, with Se—O bond lengths in the range 1.6856 (7)–1.7202 (10) Å and O—Se—O angles in the range 101.98 (3)–103.11 (5)°, and both are μ_2_-*O*:*O*-bonded to a pair of Na^+^ cations. Hydrogen bonds involving all water molecules and selenite O atoms consolidate the crystal packing. Although anhydrous Na_2_SeO_3_ and Na_2_TeO_3_ are isotypic, the title compound is surprisingly not isotypic with Na_2_TeO_3_·5H_2_O. In the tellurite hydrate, all Na^+^ cations have an octa­hedral coordination and the TeO_3_ groups are bonded to Na^+^ only *via* one of their three O atoms.

## Related literature

For the crystal structure of Na_2_TeO_3_·5H_2_O, see: Philippot *et al.* (1979 ▶). For crystal structure of anhydrous Na_2_SeO_3_ and Na_2_TeO_3_, see: Wickleder (2002 ▶); Masse *et al.* (1980 ▶). For the crystal structures of the isotypic series MgSO_3_·6H_2_O, MgSeO_3_·6H_2_O, MgTeO_3_·6H_2_O, and Mg(HPO_3_)·6H_2_O, see: Andersen & Lindqvist (1984 ▶); Andersen *et al.* (1984 ▶); Powell *et al.* (1994 ▶). For Na_2_(HPO_3_)·5H_2_O, see: Brodalla *et al.* (1978 ▶). For pharmaceutical aspects of Na_2_SeO_3_·5H_2_O, see: European Pharmacopoeia (2013 ▶). For van der Waals radii, see: Rowland & Taylor (1996 ▶).

## Experimental

### 

#### Crystal data

Na_2_SeO_3_·5H_2_O
*M* *_r_* = 263.02
Orthorhombic, 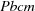

*a* = 6.5865 (2) Å
*b* = 17.2263 (6) Å
*c* = 14.7778 (6) Å
*V* = 1676.70 (10) Å^3^
*Z* = 8
Mo *K*α radiation
μ = 4.58 mm^−1^
*T* = 100 K
0.35 × 0.21 × 0.14 mm

#### Data collection

Bruker SMART CCD diffractometer
Absorption correction: multi-scan (*SADABS*; Bruker, 2003 ▶) *T*_min_ = 0.503, *T*_max_ = 0.746
23979 measured reflections
2529 independent reflections
2435 reflections with *I* > 2σ(*I*)
*R*_int_ = 0.022

#### Refinement

*R*[*F* ^2^ > 2σ(*F* ^2^)] = 0.015
*wR*(*F* ^2^) = 0.039
*S* = 1.07
2529 reflections
150 parameters
70 restraints
All H-atom parameters refined
Δρ_max_ = 0.64 e Å^−3^
Δρ_min_ = −0.58 e Å^−3^

### 

Data collection: *SMART* (Bruker, 2003 ▶); cell refinement: *SAINT* (Bruker, 2003 ▶); data reduction: *SAINT*; program(s) used to solve structure: *SHELXS97* (Sheldrick, 2008 ▶); program(s) used to refine structure: *SHELXL97* (Sheldrick, 2008 ▶); molecular graphics: *DIAMOND* (Brandenburg, 2012 ▶); software used to prepare material for publication: *SHELXL97* and *publCIF* (Westrip, 2010 ▶).

## Supplementary Material

Crystal structure: contains datablock(s) I, New_Global_Publ_Block. DOI: 10.1107/S1600536813028602/bt6939sup1.cif

Structure factors: contains datablock(s) I. DOI: 10.1107/S1600536813028602/bt6939Isup2.hkl

Additional supplementary materials:  crystallographic information; 3D view; checkCIF report

## Figures and Tables

**Table 1:** Selected bond lengths (Å)

Na1—O7*W*	2.3266 (9)
Na1—O6*W*	2.3600 (9)
Na1—O2	2.3650 (9)
Na1—O5*W*	2.3781 (10)
Na1—O4	2.4119 (9)
Na2—O9*W* ^i^	2.3458 (9)
Na2—O6*W*	2.3520 (9)
Na2—O10*W*	2.3852 (9)
Na2—O8*W*	2.3930 (9)
Na2—O7*W* ^ii^	2.4056 (9)
Na2—O9*W*	2.5108 (9)
Se1—O2	1.6857 (7)
Se1—O2^iii^	1.6857 (7)
Se1—O1	1.7164 (10)
Se2—O4	1.6856 (7)
Se2—O4^iii^	1.6856 (7)
Se2—O3	1.7202 (10)

Symmetry codes: (i) 
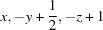
; (ii) 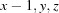; (iii) 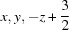.

**Table 2:** Hydrogen-bond geometry (Å, °)

*D*—H⋯*A*	*D*—H	H⋯*A*	*D*⋯*A*	*D*—H⋯*A*
O5*W*—H5*A*⋯O3^iv^	0.83 (1)	2.12 (1)	2.9468 (16)	173 (3)
O5*W*—H5*B*⋯O1^iv^	0.83 (1)	2.58 (2)	3.3631 (17)	158 (3)
O6*W*—H6*A*⋯O1	0.83 (1)	1.95 (1)	2.7704 (12)	176 (2)
O6*W*—H6*B*⋯O3	0.83 (1)	2.05 (1)	2.8660 (11)	169 (2)
O7*W*—H7*A*⋯O4^v^	0.83 (1)	1.89 (1)	2.7252 (11)	176 (2)
O7*W*—H7*B*⋯O10*W* ^v^	0.83 (1)	2.09 (1)	2.8740 (12)	157 (2)
O8*W*—H8*AB*⋯O2	0.84 (1)	1.96 (1)	2.7744 (9)	166 (1)
O9*W*—H9*A*⋯O1	0.83 (1)	1.99 (1)	2.8027 (11)	168 (2)
O9*W*—H9*B*⋯O2^ii^	0.83 (1)	1.91 (1)	2.7259 (11)	168 (2)
O10*W*—H10*A*⋯O4^v^	0.83 (1)	1.96 (1)	2.7672 (11)	164 (2)
O10*W*—H10*B*⋯O3^vi^	0.83 (1)	2.01 (1)	2.8374 (11)	174 (2)

Symmetry codes: (ii) 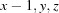; (iv) 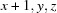; (v) 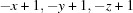; (vi) 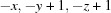.

**Fig. 1.**
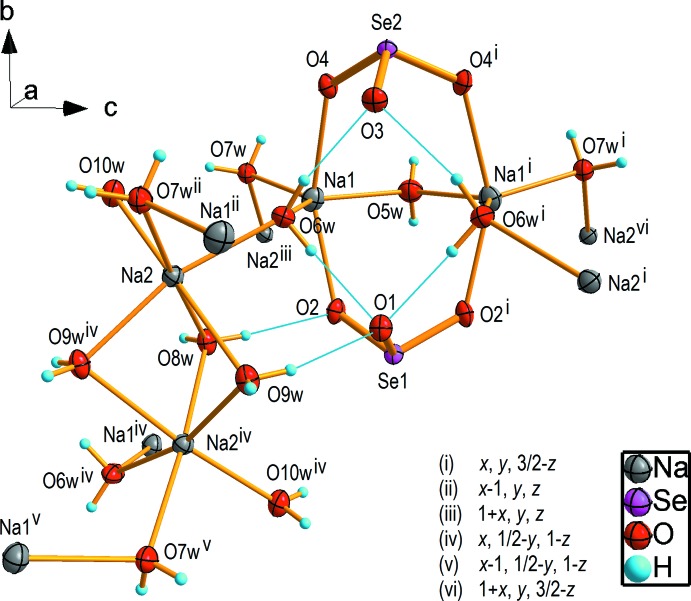
View of a characteristic part of the crystal structure of Na2SeO3.5H2O. Thermal displacement ellipsoids are shown at the 70% probability level. Symmetry operators are given on the lower right.

**Fig. 2.**
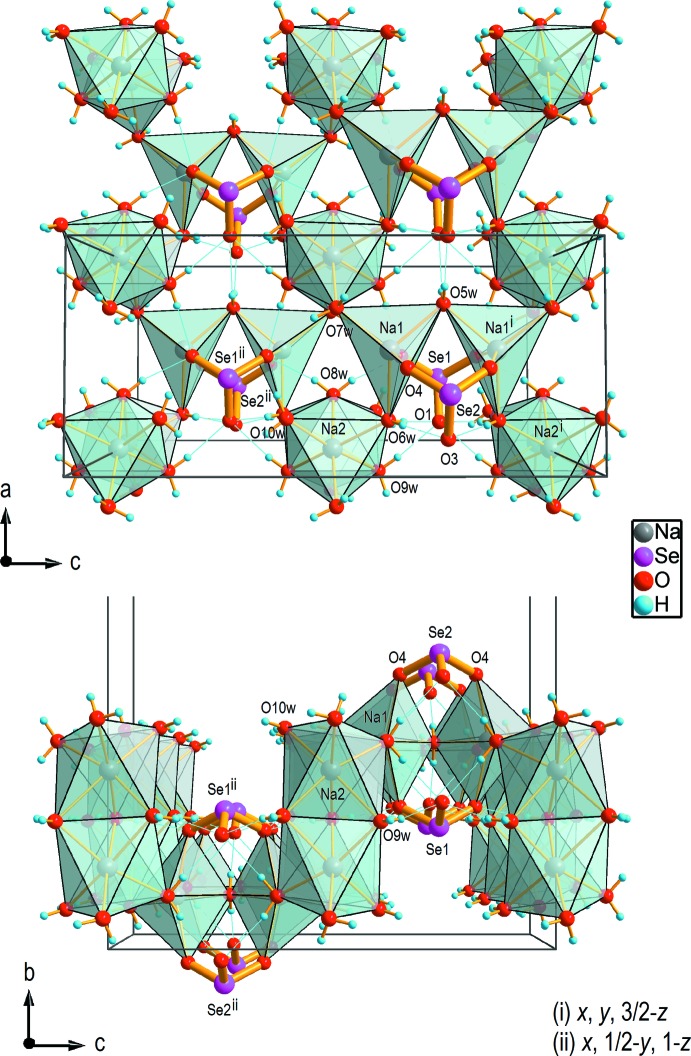
Two perspective representations of one Na2SeO3.5H2O layer parallel to (010) at y~ 1/4, viewed along [010] (top) and along [100] (bottom). Hydrogen bonds are indicated by blue lines. Symmetry operators are given on the lower right.

**Fig. 3.**
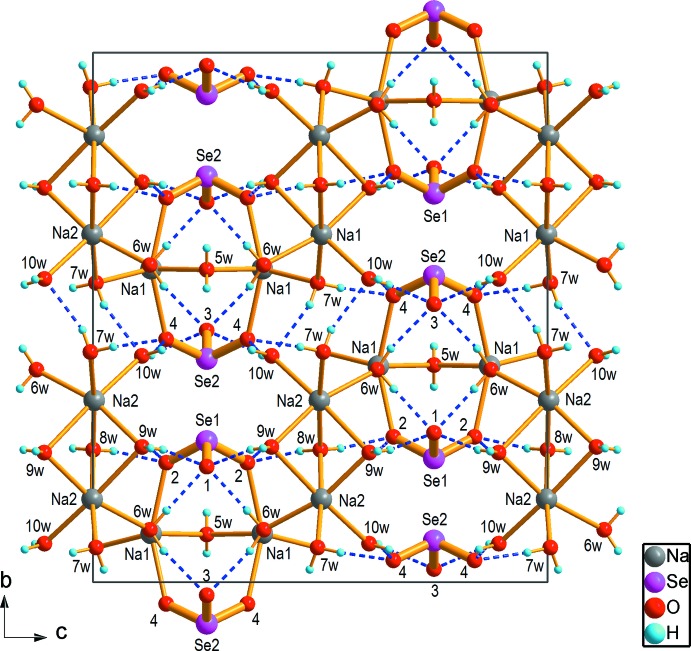
Projection of the structure of Na2SeO3.5H2O along [100] with hydrogen bonds shown as dashed lines. Symmetry codes and the letter O for oxygen atoms have been omitted for legibility.

**Fig. 4.**
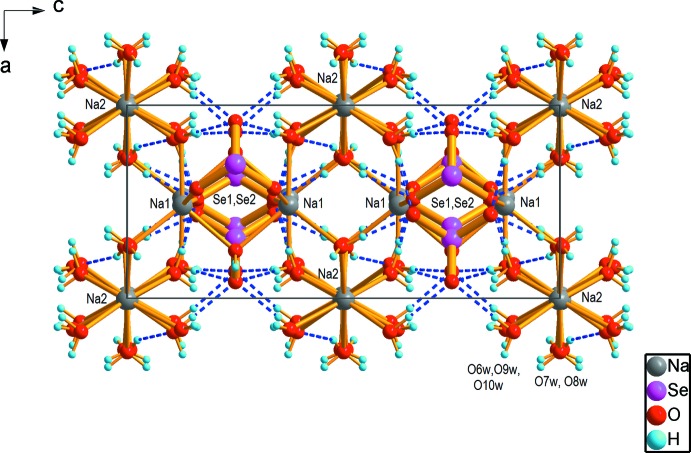
Projection of the structure of Na2SeO3.5H2O parallel to [010] with hydrogen bonds shown as dashed lines. Some coinciding sites have been labeled as guidance.

**Fig. 5.**
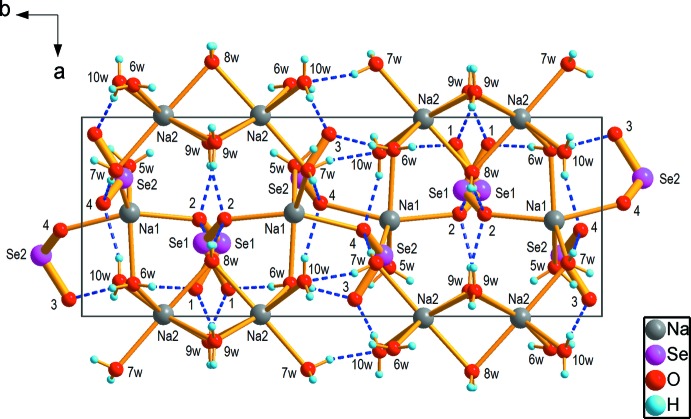
Projection of the structure of Na2SeO3.5H2O parallel to [001] with hydrogen bonds shown as dashed lines. Symmetry codes and the letter O for oxygen atoms have been omitted for legibility.

## supplementary crystallographic information

### 1. Comment

During an investigation of some simple salt hydrates the question for the crystal structure of the title compound Na_2_SeO_3_.5H_2_O arose. Although this solid is a commercial commodity, nutrition supplement, fertilizer additive, and therapeutic substance (European Pharmacopoeia, 2013), its crystallography turned out to be barren land. In order to close this gap, a crystal structure determination of the title compound was carried out.

Fig. 1 shows a characteristic part of the structure. The atoms Se1, O1, Se2, O3, O5w and its two hydrogen atoms H5a and H5b are located on a mirror plane at *x*,*y*,3/4. The water oxygen O8w is located on a twofold axis at *x*,1/4,1/2. All other atoms are in the general position. There are two independent selenite groups in the structure. Both have point symmetry *C*_s_–m and an almost undistorted trigonal pyramidal geometry with Se—O bond distances of 1.6857 (7) – 1.7202 (10) Å (mean value 1.697 (2) Å) and O—Se—O angles of 101.98 (3) – 103.11 (5)° (mean value 102.4 (5)°; Fig. 1 and Table 1). Comparable dimensions have been reported for anhydrous Na_2_SeO_3_ (Wickleder, 2002), MgSeO_3_.6H_2_O (Andersen *et al.*, 1984) and numerous other simple selenites. In the title compound the two selenite groups are bonded to a pair of mirror related Na1 atoms, which have a distorted trigonal dipyramidal coordination by three water molecules (O5w, O6w, O7w) in equatorial positions and two selenite oxygen atoms (O2 of Se1 and O4 of Se2) in apical positions. The two pentagonal bipyramids share a common corner *via* the water molecule O5w. Thus a compact group {(Na1)_2_(H_2_O)_5_(SeO_3_)_2_} is formed, which is reinforced by four internal hydrogen bonds donated by O6w and O6w^i^ to O1 and O3 (Fig. 1). The second sodium atom, Na2, has an octahedral coordination by water molecules only. This Na2(H_2_O)_6_ octahedron shares a face with a second Na2 octahedron related by a twofold axis through O8w to form a double octahedron {(Na2)_2_(H_2_O)_9_}. The two building blocks of the structure, {(Na1)_2_(H_2_O)_5_(SeO_3_)_2_} and {(Na2)_2_(H_2_O)_9_}, are then mutually linked *via* four common water molecules (two O6w and two O7w) to form corrugated layers of the composition Na_2_SeO_3_.5H_2_O extending at *y*≈ 1/4 and *y*≈ 3/4 parallel to (010). A top and a side view of a Na_2_SeO_3_.5H_2_O layer including hydrogen bonds is depicted in Fig. 2. All water molecules have approximately tetrahedral coordination figures, either by two Na and two hydrogen bond acceptors (O5w, O6w, O7w, O8w, O9w) or by one Na, one hydrogen bond donor and two hydrogen bond acceptors (O10w). Hydrogen bond data given in Table 2 show normal values for all water molecules except O5w, which has one weak and one very weak interaction (O···O = 2.9468 (16) and 3.3631 (17) Å) with the selenite oxygen atoms O1 and O3 belonging to the same Na_2_SeO_3_.5H_2_O layer. All other hydrogen bonds have O···O distances in the narrow range of 2.7252 (11) to 2.8660 (11) Å with O—H···O angles between 157 (2)° and 176 (2)°. The water molecules of O5w, O6w, O8w (has two symmetry equivalent H-bonds and therefore only one entry in Table 2), and O9w feature exclusively intra-layer hydrogen bonds. Only O7w and O10w donate four inter-layer hydrogen bonds, which explains the observed good cleavage of the crystals along (010). Figures 3, 4 and 5 show projections of the crystal structure. Fig. 3 gives a view parallel to the *a* axis, this is parallel to two Na_2_SeO_3_.5H_2_O layers. It reveals that the electron lone-pairs of Se1 and Se2 point to an open space between the layers. The shortest distance between adjacent Se atoms of two different Na_2_SeO_3_.5H_2_O layers is Se1···Se2(1 - *x*,1/2 - *y*,3/2 - *z*) = 3.438 Å and corresponds to a mutual off-set of the two Se by 2.152 Å along [100]. This Se1···Se2 distance is *ca* 0.2 Å smaller than the sum of the van der Waals radii for two Se (2 × 1.80 Å; Rowland & Taylor, 1996) and might indicate a weak mutual interaction.

The crystal structure of anhydrous Na_2_SeO_3_ is known (Wickleder, 2002). This monoclinic structure can be seen as a distorted NaCl lattice (Masse *et al.*, 1980), where Se replacing one out of three Na is shifted along a body diagonal of the NaCl lattice so that it has only three facial instead of six octahedral Se—O bonds while two Na atoms maintain their octahedral NaO_6_ coordination of the NaCl lattice. In reality the oxygen atoms are shifted more than Na and Se because they compensate arising voids and bond length differences between <Na—O> = 2.48 Å and <Se—O> = 1.70 Å. Anhydrous Na_2_TeO_3_ (Masse *et al.*, 1980) is isostructural with Na_2_SeO_3_ but is more regular because of the larger size of Te (<Te—O> = 1.88 Å) within the host lattice of the NaO_6_ octahedra with <Na—O> = 2.50 Å. Both salts furnish on crystallization from water pentahydrates, namely the title compound Na_2_SeO_3_.5H_2_O and Na_2_TeO_3_.5H_2_O. In view of the isomorphism of the anhydrous couple it is somewhat unexpected that Na_2_TeO_3_.5H_2_O does not adopt the crystal structure of the title compound or *vice versa*. The tellurite hydrate crystallizes in the monoclinic space group *C*2/c (Philippot *et al.*, 1979). It features a framework structure of the composition Na_4_(H_2_O)_10_(TeO_3_)_2_ containing three different kinds of Na. Two of these Na have relative regular octahedral coordination figures by water molecules and form infinite chains Na_3_(H_2_O)_10_≡ (H_2_O)_3_Na1(H_2_O)_3_Na2(H_2_O)_3_Na1 with two face- and one edge-sharing links per section. A third kind of Na with a strongly deformed centrosymmetric octahedral coordination by four H_2_O and two O bridges the chains *via* four corner-sharing links and carries simultaneously two TeO_3_ groups bonded to it η^1^-O-mode, *i.e. via* only one of their three O atoms. A reasonable alternative for the structures of Na_2_SeO_3_.5H_2_O and Na_2_TeO_3_.5H_2_O could be the structure of Na_2_(HPO_3_).5H_2_O, a phosphonate with a layered structure of Na(H_2_O)_5_ square pyramids, Na(H_2_O)_5_(O) octahedra, and η^1^-O-bonded HPO_3_ groups linked together only *via* shared edges and corners (Brodalla *et al.*, 1978). In it the P-bonded phosphonate H atom occupies that space which the electron lone-pair lobes of Se or Te would favour. A proof for this concept and good example for a single common structure type of four different pyramidal XO_3_^2-^ anions is the series MgSO_3_.6H_2_O (Andersen & Lindqvist, 1984), MgSeO_3_.6H_2_O (Andersen *et al.*, 1984), MgTeO_3_.6H_2_O (Andersen *et al.*, 1984), and Mg(HPO_3_).6H_2_O (Powell *et al.*, 1994) consisting of Mg(H_2_O)_6_ octahedra and XO_3_ pyramids (disregarding the H in HPO_3_) bound together by an elastic system of hydrogen bonds into a trigonal lattice of space group *R*3.

### 2. Experimental

Na_2_SeO_3_ (p.A. Merck) was dissolved in a small amount of deionized water. The solution was then slowly evaporated at T ≈ 285 K and gave after seeding colourless prsimatic crystals of Na_2_SeO_3_.5H_2_O, which were placed of filter paper in order to remove adherent mother liquor. A crystal was then immediately mounted under Paratone oil on a MiTeGen MicroLoop^TM^ and transferred to a Bruker SMART APEX diffractometer equipped with a Bruker Kryoflex cooler.

### 3. Refinement

All hydrogen atoms were clearly visible in a difference Fourier synthesis and refined satisfactorily without restraints. In the final refinement all water molecules were restrained to have similar O—H and similar intramolecular H···H distances using two SADI (σ = 0.01 Å) restraints (Sheldrick, 2008). The isotropic *U*_iso_(H) were freely refined.

### Crystal data

**Table:**

Na_2_SeO_3_·5H_2_O	*F*(000) = 1040
*M**_r_* = 263.02	*D*_x_ = 2.084 Mg m^−^^3^
Orthorhombic, *P**b**c**m*	Mo *K*α radiation, λ = 0.71073 Å
Hall symbol: -P 2c 2b	Cell parameters from 7917 reflections
*a* = 6.5865 (2) Å	θ = 2.4–30.0°
*b* = 17.2263 (6) Å	µ = 4.58 mm^−^^1^
*c* = 14.7778 (6) Å	*T* = 100 K
*V* = 1676.70 (10) Å^3^	Prism, colourless
*Z* = 8	0.35 × 0.21 × 0.14 mm

### Data collection

**Table:**

Bruker SMART CCD diffractometer	2529 independent reflections
Radiation source: fine-focus sealed tube	2435 reflections with *I* > 2σ(*I*)
Graphite monochromator	*R*_int_ = 0.022
ω and φ scans	θ_max_ = 30.0°, θ_min_ = 2.4°
Absorption correction: multi-scan (*SADABS*; Bruker, 2003)	*h* = −9→9
*T*_min_ = 0.503, *T*_max_ = 0.746	*k* = −22→24
23979 measured reflections	*l* = −20→16

### Refinement

**Table:**

Refinement on *F*^2^	Primary atom site location: structure-invariant direct methods
Least-squares matrix: full	Secondary atom site location: difference Fourier map
*R*[*F*^2^ > 2σ(*F*^2^)] = 0.015	Hydrogen site location: inferred from neighbouring sites
*wR*(*F*^2^) = 0.039	All H-atom parameters refined
*S* = 1.07	*w* = 1/[σ^2^(*F*_o_^2^) + (0.0224*P*)^2^ + 0.7143*P*] where *P* = (*F*_o_^2^ + 2*F*_c_^2^)/3
2529 reflections	(Δ/σ)_max_ = 0.003
150 parameters	Δρ_max_ = 0.64 e Å^−^^3^
70 restraints	Δρ_min_ = −0.58 e Å^−^^3^

### Special details

**Table:**

Geometry. All e.s.d.'s (except the e.s.d. in the dihedral angle between two l.s. planes) are estimated using the full covariance matrix. The cell e.s.d.'s are taken into account individually in the estimation of e.s.d.'s in distances, angles and torsion angles; correlations between e.s.d.'s in cell parameters are only used when they are defined by crystal symmetry. An approximate (isotropic) treatment of cell e.s.d.'s is used for estimating e.s.d.'s involving l.s. planes.
Refinement. Refinement of *F*^2^ against ALL reflections. The weighted *R*-factor *wR* and goodness of fit *S* are based on *F*^2^, conventional *R*-factors *R* are based on *F*, with *F* set to zero for negative *F*^2^. The threshold expression of *F*^2^ > σ(*F*^2^) is used only for calculating *R*-factors(gt) *etc*. and is not relevant to the choice of reflections for refinement. *R*-factors based on *F*^2^ are statistically about twice as large as those based on *F*, and *R*- factors based on ALL data will be even larger.

### Fractional atomic coordinates and isotropic or equivalent isotropic displacement parameters (Å^2^)

**Table:**

	*x*	*y*	*z*	*U*_iso_*/*U*_eq_	
Na1	0.51942 (7)	0.40815 (3)	0.62757 (3)	0.01543 (9)	
Na2	0.00865 (7)	0.34328 (2)	0.49775 (3)	0.01121 (8)	
Se1	0.36729 (2)	0.233898 (8)	0.7500	0.00946 (4)	
O1	0.13882 (15)	0.28182 (7)	0.7500	0.01128 (19)	
O2	0.48606 (11)	0.27444 (4)	0.66069 (5)	0.01294 (14)	
Se2	0.30596 (2)	0.578291 (8)	0.7500	0.00853 (4)	
O3	0.08542 (15)	0.52480 (6)	0.7500	0.01135 (19)	
O4	0.43189 (11)	0.54110 (4)	0.66066 (5)	0.01209 (14)	
O5W	0.7537 (2)	0.41001 (7)	0.7500	0.0182 (2)	
H5A	0.840 (3)	0.4453 (11)	0.7500	0.051 (9)*	
H5B	0.821 (4)	0.3692 (10)	0.7500	0.112 (17)*	
O6W	0.16164 (12)	0.40116 (4)	0.62475 (6)	0.01327 (15)	
H6A	0.149 (3)	0.3651 (7)	0.6612 (9)	0.030 (5)*	
H6B	0.142 (3)	0.4410 (6)	0.6550 (10)	0.038 (5)*	
O7W	0.73311 (12)	0.43467 (5)	0.50608 (5)	0.01336 (14)	
H7A	0.682 (2)	0.4442 (10)	0.4557 (8)	0.030 (4)*	
H7B	0.783 (2)	0.4762 (7)	0.5234 (10)	0.030 (4)*	
O8W	0.27783 (17)	0.2500	0.5000	0.0132 (2)	
H8AB	0.3565 (17)	0.2526 (12)	0.5444 (5)	0.035 (5)*	
O9W	−0.12523 (11)	0.24467 (5)	0.60772 (6)	0.01310 (15)	
H9A	−0.053 (2)	0.2498 (10)	0.6531 (9)	0.030 (5)*	
H9B	−0.2434 (15)	0.2472 (11)	0.6262 (11)	0.035 (5)*	
O10W	0.17400 (12)	0.42733 (5)	0.39277 (6)	0.01412 (15)	
H10A	0.2910 (15)	0.4283 (10)	0.3722 (11)	0.031 (5)*	
H10B	0.099 (2)	0.4383 (11)	0.3494 (9)	0.030 (5)*	

### Atomic displacement parameters (Å^2^)

**Table:**

	*U*^11^	*U*^22^	*U*^33^	*U*^12^	*U*^13^	*U*^23^
Na1	0.0141 (2)	0.0175 (2)	0.0148 (2)	−0.00088 (16)	0.00163 (16)	0.00129 (16)
Na2	0.01154 (18)	0.01093 (18)	0.01114 (19)	0.00028 (14)	−0.00033 (14)	−0.00044 (14)
Se1	0.00905 (7)	0.01022 (7)	0.00911 (7)	0.00145 (4)	0.000	0.000
O1	0.0073 (4)	0.0151 (5)	0.0114 (5)	0.0017 (4)	0.000	0.000
O2	0.0110 (3)	0.0179 (4)	0.0099 (3)	0.0010 (3)	0.0022 (3)	0.0016 (3)
Se2	0.00845 (7)	0.00848 (7)	0.00867 (7)	−0.00085 (4)	0.000	0.000
O3	0.0080 (4)	0.0127 (5)	0.0133 (5)	−0.0022 (4)	0.000	0.000
O4	0.0111 (3)	0.0150 (3)	0.0101 (3)	−0.0005 (3)	0.0018 (3)	−0.0014 (3)
O5W	0.0184 (5)	0.0175 (6)	0.0186 (6)	−0.0028 (5)	0.000	0.000
O6W	0.0166 (4)	0.0106 (4)	0.0126 (4)	−0.0008 (3)	−0.0011 (3)	−0.0007 (3)
O7W	0.0151 (3)	0.0140 (3)	0.0110 (3)	0.0005 (3)	−0.0004 (3)	0.0001 (3)
O8W	0.0116 (5)	0.0181 (5)	0.0099 (5)	0.000	0.000	−0.0010 (4)
O9W	0.0092 (3)	0.0189 (4)	0.0112 (3)	−0.0001 (3)	0.0004 (3)	−0.0018 (3)
O10W	0.0108 (3)	0.0183 (4)	0.0132 (4)	−0.0008 (3)	0.0001 (3)	0.0022 (3)

### Geometric parameters (Å, º)

**Table:**

Na1—O7W	2.3266 (9)	Se2—O3	1.7202 (10)
Na1—O6W	2.3600 (9)	O5W—Na1^iii^	2.3781 (10)
Na1—O2	2.3650 (9)	O5W—H5A	0.831 (10)
Na1—O5W	2.3781 (10)	O5W—H5B	0.833 (10)
Na1—O4	2.4119 (9)	O6W—H6A	0.826 (9)
Na2—O9W^i^	2.3458 (9)	O6W—H6B	0.830 (9)
Na2—O6W	2.3520 (9)	O7W—Na2^iv^	2.4057 (9)
Na2—O10W	2.3852 (9)	O7W—H7A	0.833 (9)
Na2—O8W	2.3930 (9)	O7W—H7B	0.829 (9)
Na2—O7W^ii^	2.4056 (9)	O8W—Na2^i^	2.3930 (9)
Na2—O9W	2.5108 (9)	O8W—H8AB	0.837 (8)
Se1—O2	1.6857 (7)	O9W—Na2^i^	2.3457 (9)
Se1—O2^iii^	1.6857 (7)	O9W—H9A	0.825 (9)
Se1—O1	1.7164 (10)	O9W—H9B	0.826 (9)
Se2—O4	1.6856 (7)	O10W—H10A	0.828 (9)
Se2—O4^iii^	1.6856 (7)	O10W—H10B	0.829 (9)
			
O7W—Na1—O6W	126.90 (3)	Na1—O5W—Na1^iii^	99.06 (5)
O7W—Na1—O2	114.03 (3)	Na1—O5W—H5A	116.9 (10)
O6W—Na1—O2	82.02 (3)	Na1^iii^—O5W—H5A	116.9 (10)
O7W—Na1—O5W	101.06 (4)	Na1—O5W—H5B	109.6 (13)
O6W—Na1—O5W	131.46 (4)	Na1^iii^—O5W—H5B	109.6 (13)
O2—Na1—O5W	85.18 (4)	H5A—O5W—H5B	104.6 (15)
O7W—Na1—O4	96.58 (3)	Na2—O6W—Na1	117.62 (4)
O6W—Na1—O4	79.24 (3)	Na2—O6W—H6A	99.2 (11)
O2—Na1—O4	149.39 (3)	Na1—O6W—H6A	97.2 (12)
O5W—Na1—O4	89.32 (4)	Na2—O6W—H6B	135.6 (12)
O9W^i^—Na2—O6W	164.81 (3)	Na1—O6W—H6B	95.9 (13)
O9W^i^—Na2—O10W	97.56 (3)	H6A—O6W—H6B	104.8 (12)
O6W—Na2—O10W	93.79 (3)	Na1—O7W—Na2^iv^	111.55 (3)
O9W^i^—Na2—O8W	81.60 (3)	Na1—O7W—H7A	119.0 (12)
O6W—Na2—O8W	87.49 (3)	Na2^iv^—O7W—H7A	112.8 (12)
O10W—Na2—O8W	94.49 (3)	Na1—O7W—H7B	99.9 (12)
O9W^i^—Na2—O7W^ii^	99.97 (3)	Na2^iv^—O7W—H7B	106.4 (12)
O6W—Na2—O7W^ii^	90.29 (3)	H7A—O7W—H7B	105.4 (12)
O10W—Na2—O7W^ii^	88.88 (3)	H8AB^i^—O8W—Na2^i^	115.7 (11)
O8W—Na2—O7W^ii^	176.07 (3)	H8AB^i^—O8W—Na2	118.9 (12)
O9W^i^—Na2—O9W	82.01 (3)	Na2^i^—O8W—Na2	84.39 (4)
O6W—Na2—O9W	85.46 (3)	H8AB^i^—O8W—H8AB	103.5 (14)
O10W—Na2—O9W	172.76 (3)	Na2^i^—O8W—H8AB	118.9 (12)
O8W—Na2—O9W	78.28 (3)	Na2—O8W—H8AB	115.7 (11)
O7W^ii^—Na2—O9W	98.32 (3)	Na2^i^—O9W—Na2	82.82 (3)
O2^iii^—Se1—O2	103.06 (5)	Na2^i^—O9W—H9A	113.2 (12)
O2^iii^—Se1—O1	101.98 (3)	Na2—O9W—H9A	104.5 (12)
O2—Se1—O1	101.98 (3)	Na2^i^—O9W—H9B	127.4 (12)
Se1—O2—Na1	127.49 (4)	Na2—O9W—H9B	120.6 (13)
O4^iii^—Se2—O4	103.11 (5)	H9A—O9W—H9B	105.5 (12)
O4^iii^—Se2—O3	102.24 (3)	Na2—O10W—H10A	132.5 (12)
O4—Se2—O3	102.24 (3)	Na2—O10W—H10B	111.7 (12)
Se2—O4—Na1	129.59 (4)	H10A—O10W—H10B	105.2 (12)

Symmetry codes: (i) *x*, −*y*+1/2, −*z*+1; (ii) *x*−1, *y*, *z*; (iii) *x*, *y*, −*z*+3/2; (iv) *x*+1, *y*, *z*.

### Hydrogen-bond geometry (Å, º)

**Table:**

*D*—H···*A*	*D*—H	H···*A*	*D*···*A*	*D*—H···*A*
O5*W*—H5*A*···O3^iv^	0.83 (1)	2.12 (1)	2.9468 (16)	173 (3)
O5*W*—H5*B*···O1^iv^	0.83 (1)	2.58 (2)	3.3631 (17)	158 (3)
O6*W*—H6*A*···O1	0.83 (1)	1.95 (1)	2.7704 (12)	176 (2)
O6*W*—H6*B*···O3	0.83 (1)	2.05 (1)	2.8660 (11)	169 (2)
O7*W*—H7*A*···O4^v^	0.83 (1)	1.89 (1)	2.7252 (11)	176 (2)
O7*W*—H7*B*···O10*W*^v^	0.83 (1)	2.09 (1)	2.8740 (12)	157 (2)
O8*W*—H8*AB*···O2	0.84 (1)	1.96 (1)	2.7744 (9)	166 (1)
O9*W*—H9*A*···O1	0.83 (1)	1.99 (1)	2.8027 (11)	168 (2)
O9*W*—H9*B*···O2^ii^	0.83 (1)	1.91 (1)	2.7259 (11)	168 (2)
O10*W*—H10*A*···O4^v^	0.83 (1)	1.96 (1)	2.7672 (11)	164 (2)
O10*W*—H10*B*···O3^vi^	0.83 (1)	2.01 (1)	2.8374 (11)	174 (2)

Symmetry codes: (ii) *x*−1, *y*, *z*; (iv) *x*+1, *y*, *z*; (v) −*x*+1, −*y*+1, −*z*+1; (vi) −*x*, −*y*+1, −*z*+1.
